# Endoscopic inguinal lymphadenectomy for vulvar cancer, is it feasible?

**DOI:** 10.1097/CCO.0000000000001174

**Published:** 2025-07-22

**Authors:** Giacomo Guidi, Angelica Naldini, Giorgia Garganese, Simona Maria Fragomeni, Anna Fagotti, Nicoló Bizzarri

**Affiliations:** a UOC Ginecologia Oncologica, Dipartimento di Scienze Della Salute Della Donna, Del Bambino e di Sanità Pubblica, Fondazione Policlinico Universitario A. Gemelli, IRCCS, Rome; b Università Cattolica del Sacro Cuore, Rome, Italy

**Keywords:** complications, inguinal lymphadenectomy, laparoscopic surgery, lymphatic, robotic surgery, vulvar cancer, wound

## Abstract

**Purpose of review:**

The evaluation of lymph node status is crucial for the correct staging of patients with vulvar carcinoma and to assess whether adjuvant treatment is necessary. Classically, this procedure is performed with an open approach to the inguinal region; however, this procedure is associated with a significant complication rate.

For this reason, the video-endoscopic approach to inguinal lymph node assessment has been proposed in recent years. This review aimed to provide an overview on the feasibility and outcomes of the video-endoscopic inguinal lymphadenectomy (VEIL).

**Recent findings:**

VEIL appeared to be a viable alternative to the open approach, with similar outcomes in terms of the number of lymph nodes removed and blood loss. While the postoperative complications rate was lower in the VEIL group, operative times tended to be longer. This may be due to the lack of standardization of the technique and to the variability in video-endoscopic approaches (laparoscopic vs robotic). Sentinel lymph node biopsy with endoscopic approach has also been described.

**Summary:**

VEIL appears to be feasible and safe, adding benefits in terms of postoperative complications. Nevertheless, few studies are available and further data is needed to confirm these findings.

## INTRODUCTION

Cancer of the vulva is relatively rare, with 17 651 new cases recorded in 2022 in Europe; of these, 13 384 cases occurred in women aged >65aa, showing that this disease mainly involves older women [1]. The most frequent histotype is squamous cell, while the most important risk factors are age, HPV infection, smoking, immunodepression, lichen sclerosus and vulvar intraepithelial neoplasia [2].

In vulvar cancer, surgical margins and lymph node metastasis are the two most important prognostic factors for recommending adjuvant therapy [3].

For this reason, the assessment of lymph node status by inguinal lymphadenectomy and/or sentinel lymph node (SLN) biopsy is crucial for appropriate disease staging. The SLN procedure is indicated in patients with a unifocal primary tumor <4 cm with a depth invasion >1 mm but no suspicious lymph nodes at preoperative imaging. On the other hand, inguinal lymphadenectomy is indicated in patients with ≥4 cm tumor and in multifocal disease [4^▪▪^]. Moreover, after the recent publication of GROINS-V II study, the role of inguinofemoral lymphadenectomy has become more relevant in presence of SLN macrometastasis [5]. Several studies have aimed to reduce the morbidity associated with systematic lymphadenectomy. For example, the GROINS-V II trial demonstrated that, in cases of SLN micrometastasis, inguinofemoral lymphadenectomy could be safely omitted and replaced by radiotherapy [5]. Similarly, the GROSNAPET study explored the possibility of expanding eligibility for SLN biopsy to all patients accurately selected as node-negative at preoperative assessment [6].

Since the affected population is old, and therefore more frail, special attention has been paid to reducing the morbidity of surgery and possible related complications while maintaining the necessary oncological radicality.

Standard technique to inguinal lymphadenectomy and more in general to lymph node assessment (including SLN) is classically reported by open approach [4^▪▪^]. Nevertheless, with the advancement in surgical techniques, a minimally invasive (video-endoscopic) approach has been proposed.

The present review aims to provide an overview on the current role of video endoscopic inguinal lymphadenectomy (VEIL) in vulvar cancer, focusing on the studies published in English language in the last five years. Furthermore, future perspectives regarding its application have also been delineated. 

**Box 1:**
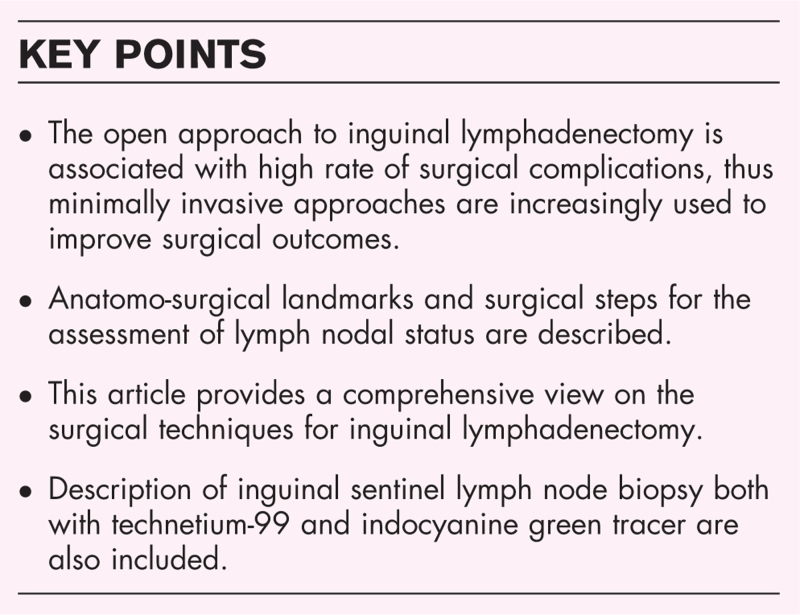
no caption available

## SURGICAL TECHNIQUES

Historically, inguinal lymph node dissection (ILND) was conducted through an open incision (O-ILND), leading to high rate of complications, reported between 50% and 90%, significantly affecting quality of life and potentially restricting the use of recommended ILND, particularly in frail patients [7^▪▪^]. For this reason, VEIL and endoscopic SLN mapping, have been developed in recent years in order to reduce postoperative complications, improve esthetic aspect and quality of life [7^▪▪^].

### Minimally invasive techniques

The VEIL consists of a minimally invasive technique to access the groin nodes and dissect them avoiding larger groin incisions. SLN technique involves the injection of a tracer, radioactive (technetium-99) with or without dye (indocyanine green – ICG) next to the vulvar lesion and with subsequent dissection of the mapped lymph node [8]. The use of radioactive tracer is still mandatory and considered the standard of care for SLN detection. At the same time ICG can be easily associated with the technetium-99 making the SLN dissection easier being guided by the green dye. The use of ICG appears to be a promising approach for its easy application and its better efficacy [4^▪▪^]; the ICG is injected subcutaneously next to the tumor site, near-infrared modality is used to detect fluorescent nodes. SLN is mapped bilaterally for central tumors and unilaterally for lateral tumors.

### Multi-port video endoscopic inguinal lymphadenectomy

**FIGURE 1:**
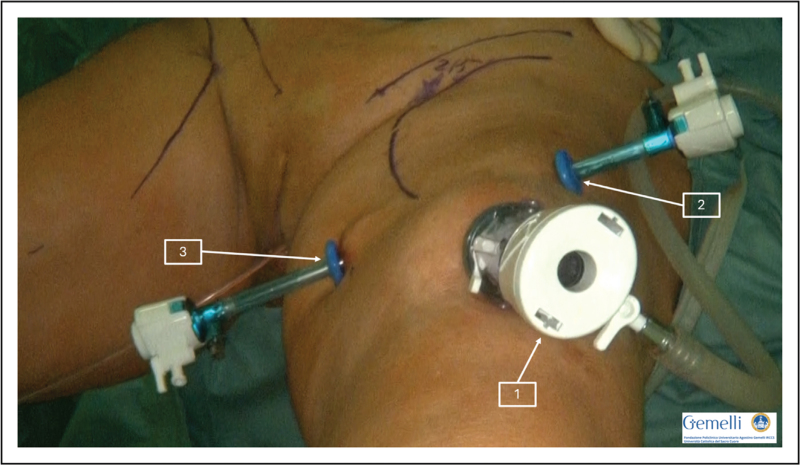
(Trocar placement): 10–12 mm port (1) is placed 3 cm below the apex of the femoral triangle (camera access point). Two ancillary 5 mm trocars (2–3) are placed medially and laterally at 3 cm from the edges of the femoral triangle.

A technique involving three incisions is typically employed (Fig. 1).

A 12 mm incision is made about 3 cm below the apex of the femoral triangle, where a 10–12 mm port is inserted to serve as the camera access point. Blunt finger dissection is used to develop the anatomical space above Scarpa's fascia. This space is completely developed by insufflation after trocar insertion.

Subsequently, two ancillary 5 mm trocars are placed approximately 3 cm of the medial and lateral edges of the femoral triangle. Extra caution is crucial at this phase to avoid injuring the femoral vessels.

The procedure begins by creating a space between the tissue containing the lymph nodes and the skin flaps.

It is important to avoid Camper's fascia dissection, as this could injure the vessels that supply the skin flaps; the loss of these vessels could lead to postoperative skin flap necrosis.

The limits of lymphatic dissection are the same as per open technique (adductor longus muscle medially, the sartorius muscle laterally, 2 cm above the inguinal ligament superiorly, Scarpa's fascia superficially, and the femoral vessels deeply).

During this dissection, lymphatic vessels should be sealed to minimize postoperative lymphorrhea. The saphenous vein is encountered at the apex of the femoral triangle and, when surgically feasible, should be preserved.

Dissection proceeds from the distal to the proximal direction, and the femoral vessels are generally encountered at this stage.

Subsequently, the femoral vessels are carefully dissected and skeletonized.

The dissection continues infero-medially, from the saphenofemoral junction, allowing for the removal of the deep inguinal lymph nodes located on the pectineus muscle. Dissection continues until the pectineus muscle is visible.

Finally, the lymph node specimen is fully detached by dissection of any remaining fascial connections to the inguinal ligament.

VEIL may be performed using two routes: VEIL through the subcutaneous route of the lower extremities (VEIL-L) and VEIL through the subcutaneous route of the hypogastric area (VEIL-H). The anatomical limits for lymph node dissection are the same as those used for the open and video-endoscopic technique described, except for the dissection that proceeds cranio-caudally in VEIL-H.

A systematic review compared the operation time, intraoperative blood loss, and time of drainage of the VEIL-L procedure seemed less than those of the VEIL-H procedure, but VEIL-L and VEIL-H were equivalent in the efficacy of lymph node dissection, surgery-related complications, and cancer recurrence rate [9].

### Single-site video-endoscopic inguinal lymphadenectomy

An alternative technique known as the laparoendoscopic single-site (LESS) inguinal lymphadenectomy has also been described [10,11^▪^].

This technique consists in a 2.5 cm incision made below the apex of the femoral triangle, similar to the location of the 12 mm laparoscope port in the multiport approach, with the placement of a single multiaccess port. This port allows for the introduction of both working instruments and the laparoscope, in order to spare the two separate ports for the left- and right-hand instruments.

### Robotic-assisted video endoscopic inguinal lymphadenectomy

Robotic approach can also be employed for VEIL. Skin incision for camera placement is made approximately 25 cm under to the central part of the inguinal ligament. Then, with blunt dissection, the plane above Scarpa's fascia is developed. This access is used for the camera port.

After placement of the camera, insufflation starts and the other two ports are placed under direct visualization; usually the incision is made 8 cm medial and lateral to the camera port, meanwhile the assistant port is placed between the lateral robotic port and camera port. The robotic system is docked at the contralateral side of the patient and, depending on the robotic system, it may need to be docked on the opposite side when switching to the other groin [12–13].

### Video assisted sentinel lymph node biopsy

SLN biopsy can also be performed by a video assisted technique in selected patients. Same tracer as per open approach can be used. However, majority of studies report the use of indocyanine green (ICG), which is associated with 99m technetium (injected the day before procedure), considered the standard of care.

**FIGURE 2:**
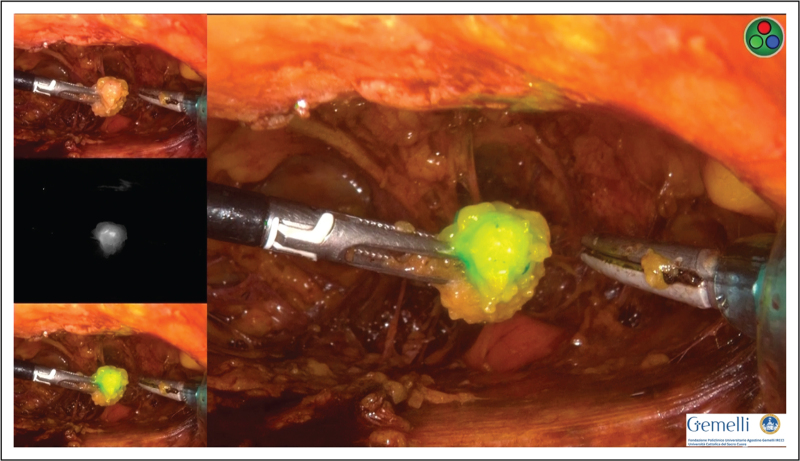
Inguinal SLN dissected. After the removal of the ICG-SLN, Gamma probe is used to confirm the concordance between ICG and 99m technetium.

The procedure is performed by blunt dissection up to the inguinal ligament isolating and preserving the lymphatic structures, particularly before the identification of SLN. During dissection, SLN is visualized with near-infrared camera and then dissected (Fig. 2). After the removal of the ICG-SLN, Gamma probe is used to confirm the concordance between ICG and 99m technetium. [14^▪▪^].

Recently, the technique on how to perform bilateral SLN biopsy using ICG by VEIL has been described [15^▪▪^].

### Postoperative management

Postoperative drains after systematic inguinofemoral lymph node (IFLN) dissection are used routinely and often left in situ until producing <20 to 50 ml per 24 h, with some recommendations to leave the drains in place at least 5–7 days postoperatively [16].

Radical vulvectomy seems to have a rate of surgical site infections (SSI) comparable with that of patients undergoing abdominal hysterectomy [17].

There is no high-quality evidence supporting antimicrobial prophylaxis in this setting. However, given the high rate of SSI, it seems reasonable to give a single dose of antibiotic, consistent with other published guidelines [18].

## OUTCOMES

Data comparing the O-ILND and VEIL in vulvar cancer are limited, and the highest quality data usually come from systematic reviews that also include penile cancer and melanoma studies. In particular, two recent reviews reported a significant difference in the occurrence of postoperative complications.

Cacciamani *et al.* reported fewer cutaneous adverse events, and infections comparing O-ILND vs. VEIL/R-VEIL, but also fewer functional and lymphatic AEs even if not statistically significant [19].

Di Donna, *et al.* reported in a systematic review and meta-analysis that VEIL was associated with a significantly lower rate of lymphatic and wound complications with only the 12.6% of VEIL groins developed a major complication after surgery versus the 41% of the O-ILND approach. In the analysis, the most common lymphatic (lymphorrea, lymphedema and lymphoceles) and wound complications (wound inguinal dehiscence, groins infection and cellulitis) were evaluated. However regardless of the type of complication, it was observed that VEIL was associated with a significantly lower morbidity rate compared with O-ILND [7^▪▪^].

Similar results were reported by Reza Nabavizadeh, *et al.*, concluding that operative time was the only variable in favor of O-ILND, even in patients without bulky groin nodal metastasis. In the same study, there were no significant difference in the number of harvested nodes and inguinal node recurrence [20].

## CONCLUSION

ILND is an essential step for staging and treatment of vulvar cancer. O-ILND is usually associated with high rate and severity of postoperative complications. VEIL represents a promising technique with reported reduced short and long-term morbidity.

Currently, there is no level I evidence comparing VEIL with traditional open approach for vulvar cancer, but these minimally invasive approach appears to be feasible, safe and reproducible, including as approach to SLN biopsy. Even if early reports on VEIL techniques have demonstrated similar oncologic outcomes to the traditional open technique with lower morbidity, further investigation by prospective studies are needed.

## Acknowledgements

*We would like to express our profound gratitude to Prof. Giovanni Scambia for his teachings and scientific inspiration.*

### Financial support and sponsorship

*None.*

### Conflicts of interest

*There are no conflicts of interest.*

## REFERENCES AND RECOMMENDED READING

Papers of particular interest, published within the annual period of review, have been highlighted as:

▪ of special interest
▪▪ of outstanding interest
